# Illumina MiSeq Sequencing for Preliminary Analysis of Microbiome Causing Primary Endodontic Infections in Egypt

**DOI:** 10.1155/2018/2837328

**Published:** 2018-04-03

**Authors:** Sally Ali Tawfik, Marwa Mohamed Azab, Ali Abdellah Abdelrahman Ahmed, Dalia Mukhtar Fayyad

**Affiliations:** ^1^Department of Microbiology & Immunology, Faculty of Pharmacy, Suez Canal University, Ismailia, Egypt; ^2^Department of Endodontics, Faculty of Dentistry, Suez Canal University, Ismailia, Egypt

## Abstract

The use of high throughput next generation technologies has allowed more comprehensive analysis than traditional Sanger sequencing. The specific aim of this study was to investigate the microbial diversity of primary endodontic infections using Illumina MiSeq sequencing platform in Egyptian patients. Samples were collected from 19 patients in Suez Canal University Hospital (Endodontic Department) using sterile # 15K file and paper points. DNA was extracted using Mo Bio power soil DNA isolation extraction kit followed by PCR amplification and agarose gel electrophoresis. The microbiome was characterized on the basis of the V3 and V4 hypervariable region of the 16S rRNA gene by using paired-end sequencing on Illumina MiSeq device. MOTHUR software was used in sequence filtration and analysis of sequenced data. A total of 1858 operational taxonomic units at 97% similarity were assigned to 26 phyla, 245 families, and 705 genera. Four main phyla Firmicutes, Bacteroidetes, Proteobacteria, and Synergistetes were predominant in all samples. At genus level,* Prevotella*,* Bacillus*,* Porphyromonas*,* Streptococcus*, and* Bacteroides* were the most abundant. Illumina MiSeq platform sequencing can be used to investigate oral microbiome composition of endodontic infections. Elucidating the ecology of endodontic infections is a necessary step in developing effective intracanal antimicrobials.

## 1. Introduction

The human microbiome is determined as the ecological community of commensal, symbiotic, and pathogenic microorganisms that actually share our body space [1, 2]. The oral microbiome is associated with the development of oral diseases such as dental caries, periodontal disease, and endodontic infection [3, 4]. Microorganisms that initially invade and colonize the pulp can lead to primary endodontic infection which is characterized by a polymicrobial infection dominated by anaerobic bacteria [5, 6]. On the other hand, secondary (persistent) infection refers to microorganisms that were members of a primary or secondary infection and that, in some way, resisted intracanal antimicrobial procedures and were able to survive during periods of nutrient deprivation in treated canals [7]. It has been reported that the bacterial communities in primary endodontic infections were more diverse than those in persistent infections [7, 8]. Apical periodontitis refers to inflammatory disease of apical periradicular tissues caused by microbial infection within the root canal system of the implicated tooth. Apical periodontitis is mainly the consequence of dental caries when the root canal system is infected by oral microbiota [9, 10]. Since the etiology and pathogenesis of apical periodontitis have not been finally elucidated, additional research should be conducted in this field.

Previously, bacterial diversity of the infected root canal system was determined by broad-range polymerase chain reaction followed by cloning and Sanger sequencing as well as molecular fingerprinting techniques such as denaturing gradient gel and terminal restriction fragment length polymorphism analysis [5, 11]. These techniques offered initial insights into the bacterial diversity but despite their high sensitivity these methods could detect only the most prevalent bacterial community members. Low abundant species may occupy critical niches within a complex microbial community and thus are potentially important in maintaining the stability and virulence of a microbial community [12].

Microbial diversity related to the human body is much greater than previously thought [13, 14]. Molecular methods have demonstrated a higher complexity of the endodontic microbiota than previously reported by cultivation approaches. Recent studies have moved away from the concept that a single pathogen causes a disease to the concept that the whole community is responsible for the pathogenicity [10, 15]. Molecular biology methods have facilitated the identification of specific bacterial species with apical periradicular disease which led to the discovery of novel endodontic pathogens [6]. Next generation sequencing has been widely used for bacterial diversity analyses using 16S ribosomal RNA gene [16]. The use of high throughput technology enables a large number of reads in a single run, providing increased sampling depth compared with other techniques [17]. The major advantage of next generation sequencing is the detection of low abundant genera [17, 18].

The aim of this study was to investigate bacterial diversity in primary endodontic infections through taxonomic classification of 16S rRNA gene by Illumine MiSeq sequencing platform. Therefore, the recognition of community profiles involved in endodontic disease with periapical lesion may represent an important step toward a better understanding of the pathogenesis of the disease and establishment of more effective therapeutic protocols.

## 2. Material and Methods

### 2.1. Subjects and Patients

Samples were obtained from 19 adult patients (15 females and 4 males; aged 18 to 51 years; mean age 31 years) who visited Suez Canal University Dental Hospital for the endodontic treatment of primary endodontic infections (untreated root). They were recruited as study subjects (10 with upper incisor, 1 with lower incisor, 6 with upper premolar, and 2 with upper molar). Patients who participated in this study were volunteers. All patients were aware about the nature of the study. They were included into the study after signing an ethics committee-approved informed consent form. This study was carried out in accordance with the guidelines of, and after approval by, the Ethic Committee of Human Research at Faculty of Pharmacy, Suez Canal University, Egypt (reference number: 201611MH1). Only teeth from adult patients with carious lesions, necrotic pulps, and radiographic evidence of apical periradicular disease were involved in this study. We excluded patients who had systemic disease, cancer, diabetes, immunodeficiency disorder, and a history of using antibiotics or fluoride in the previous 3 months [19, 20].

### 2.2. Microbiome Sample Collection

At the beginning, the tooth crown was cleansed with pumice; then local anesthesia was applied and a rubber dam was placed. 30% hydrogen peroxide was used to clean the tooth and the surrounding field which were then decontaminated with a 2.5% sodium hypochlorite solution (NaOCl) for 30 seconds each. Complete access preparations were carried out using sterile burs without water spray. 2.5% NaOCl was again used to swab the operative field including the pulp chamber. The NaOCl solution was inactivated with 5% sodium thiosulfate [21]. Little amount of sterile saline solution was inserted into the canal in case the root canal was dry. Samples were firstly collected by means of #15 K-type file with the handle cut-off. The file was inserted to a level approximately 1 mm short of the tooth apex with a gentle filing motion that depended on the diagnostic radiographs. Then, two sequential paper points were introduced to the same level and used to soak up the fluid in the canal. Each paper point was kept in position for 1 minute. The two paper points and the file were both transferred to 1.5 ml microcentrifuge tube containing 1 ml of lysis Mo Bio buffer. Afterwards, the samples were transferred to the laboratory for DNA extraction in frozen ice packs. A negative control (sample code 19) using sterile file and paper points which were not applied to the root canal was used in parallel to test for the existence of contaminants in the file and paper points [19, 22, 23].

### 2.3. DNA Extraction

Microcentrifuge tubes containing endodontic samples were vortexed at low speed to disperse the trapped bacteria. Genomic DNA from samples and the negative control were extracted immediately after sample collection using Mo Bio power soil DNA isolation kit with some modifications (Mo Bio Laboratories, Carlsbad, CA, USA, cat. number 12888-50). The file and paper points were removed and the solution was transferred to bead tubes. Then, 60 *μ*L of solution C1 had been added to the bead tubes. Using water bath, tubes were incubated at 65°C for 10 minutes and then vortexed horizontally at maximum speed for 2 minutes using a flat-bed vortex pad with tape at maximum speed for 10 minutes. Afterwards, instructions were followed as directed by the manufacturer protocol except eluting DNA with 30 *μ*L instead of 100 *μ*L of PCR DNase-free water. DNA samples were measured using a Nanodrop ND-1000 spectrophotometer (ND-1000; Thermo Scientific, Waltham, MA, USA), by measuring absorbance values at 260 and 280 nm.

### 2.4. PCR Amplification

PCR amplification was carried out immediately after DNA extraction, using primers targeting the V3 and V4 regions of the 16S rRNA gene which used the extracted DNA as a template. Hypervariable regions V3-V4 of 16S rRNA gene were amplified using the following primers with Illumina adapters (underlined): forward primer: 5′TCGTCGGCAGCGTCAGATGTGTATAAGAGACAGCC­TACGGGNGGCWGCAG; reverse primer: 5 GTCT­CGTGGGCTCGGAGATGTGTATAAGAGACAGGACTA­CHVGGGTATCTAATCC [24].

The PCR mixture was composed of 0.8 *μ*L for each forward and reverse primer (10 *μ*M, Metabion, Germany), 3 *μ*L of template DNA for the samples, and 12.5 *μ*L of 1x of Hot Master Mix (Promega GoTaq® Green Master Mix) to a final volume of 25 *μ*L. For negative control, 3 *μ*L of elution solution was used. The amplifications were performed under the following conditions: initial denaturation at 95°C for 2 minutes, followed by 30 cycles of denaturation at 95°C for 30 seconds, primer annealing at 60°C for 30 seconds, and extension at 72°C for 30 seconds, with a final elongation at 72°C for 5 minutes. The presence of PCR products was confirmed by electrophoresis in a 2% agarose gel conducted at 80 V/cm in Tris-Borate-EDTA (TBE) buffer. Ethidium bromide (0.5 lg/ml) was used to stain the gel for 15 min which is then visualized under 300 nm ultraviolet light. The resulting image was then captured using a computer software program (AlphaEase™, Alpha lnnotech). PCR amplicons that were positive on the agarose gel electrophoresis images were subjected to purification by means of the MinElute kit (QIAGEN). The purified DNA products were pooled together according to equal concentrations where short fragments were removed using Ampure beads (Agencourt Bioscience, MA, USA). The eluted DNA product obtained after purification was quantified using Qubit Kit Assays (Invitrogen, Life Technologies). Bioanalyzer 2100 with the DNA 1000 Chip kit (Agilent, Palo Alto, CA, USA) was used to assess the quality of the final products for each sample individually [19, 24]. There was no amplification product observed in the negative extraction controls.

### 2.5. Illumina Sequencing of 16S rRNA Gene

Sequencing was carried out at IGA Technology Services (Udine, Italy) using paired-end Illumina MiSeq sequencing on an Illumina MiSeq device (Illumina Inc., San Diego, CA, USA) with 600 cycles (300 cycles for each paired read and 12 cycles for the barcode sequence) according to the manufacturer's instructions. To artificially increase the genetic diversity, it has been common to mix in a control library of genomic DNA from the phage phix to prevent focusing and phasing problems due to the sequencing of “low diversity” libraries. Sequence analysis was conducted using the 16S-based metagenomics workflow of MiSeq Reporter v2.3 (Illumina). 16S rRNA gene is the widely used target to identify microbes without the need to sequence entire genome. Illumina workflow started with purified genomic DNA. Primers were tailed with sequences to include indexing barcodes. Samples were gathered into a single library for sequencing on Illumina MiSeq sequencing system which generated paired 300 bp reads. Sequences were then demultiplexed based on index sequences. FASTQ files with Quality Score Encoding were created. OTUs clustering and classification at several taxonomic levels, kingdom, phylum, class, order, family, genus, and species, were performed. Illumina-curated version of the Greengenes database was used as a taxonomy database for the metagenomics workflow (http://greengenes.secondgenome.com/downloads/database/13_5).

### 2.6. Analysis of Microbial Community with MOTHUR

Analysis of sequenced data was done by using MOTHUR software (v.1.38.1) and the pipeline adapted from standard operating procedure (SOP) from Schloss et al. [25]. The two sets of reads for each sample (forward and reverse reads) were combined using (make.contigs) command. Sequences that failed to fulfil any one of the following criteria were excluded: maximum length of 600 bases, the presence of any ambiguities, maximum homopolymer length of 8 nt, and more than 1 nucleotide mismatch to the primer using (screen.seqs) command. Duplicated sequences were removed using (unique.seqs) command [25, 26]. SILVA database was customized to the targeted V3-V4 region of 16S rRNA gene using the (pcr.seqs) command which corresponds to the similar region in* Escherichia coli *starting from 6426 and ending at 27645. Unique sequences were aligned in MOTHUR using SILVA reference database [27]. Columns in the alignment that only contain gap characters were pulled out without losing any information using (filter.seqs) command. Then, sequences were preclustered using (pre.cluster) command which could permit at most two differences between sequences. Chimeras were detected using UCHIME algorithm and then removed using (chimera.uchime) command [28]. Sequences were taxonomically classified using the Naive Bayesian classification with 80% confidence threshold by using (classify.seqs) command [29]. Sequences that were not classified to any one of the domains (unknown) or classified in Chloroplast, Mitochondria, Eukaryota, and Archaea were eliminated using (remove.lineage) command. Sequences were analyzed from more than one point of view. Firstly, we considered each sample as a separate community. Secondly, samples were combined into two groups according to sex (males and females). Thirdly, samples were grouped into four groups according to age. Groups 1, 2, 3, and 4 ranged within 18–20, 21–30, 31–40, and 41–50 years, respectively. Lastly, based on 16S rRNA dendrogram three separate groups were constructed according to community structure and population compositions (Group A, Group B, and Group C) using (tree.shared) command which was visualized using FigTree program (v1.4.3).

### 2.7. Phylotypes Based Analysis

Sequences were analyzed into phylotypes according to their taxonomic classification using (phylotype) command. A distance matrix was created and the sequences were clustered into operational taxonomic units (OTUs) at 3% dissimilarity cut-off (97% similarity) using (dist.seqs) and (cluster) command, respectively. The cut-off numbering of the phylotypes equals 4 which corresponded to order level used in (cluster.split) command. The number of sequences in each OTU was determined using (make.shared) command. The taxonomy for each OTU was specified using (classify.otu) command. To equalize read sizes of the samples for the comparison of read sizes among the samples, random subsampling was performed (i.e., 2,000) using (sub.sample) command [25].

### 2.8. Operation Taxonomic Unit (OTU) Based Analysis

#### 2.8.1. Alpha Diversity

Rarefaction curves describe the number of OTUs observed as a function of sampling effort which were performed using (rarefaction.single) command. Community richness indices, number of observed OTUs, Chao 1 richness estimate, abundance based coverage richness estimate, and Jackknife estimator, were calculated. Community diversity indices, Simpson diversity index, inverse Simpson index, Shannon diversity index, nonparametric estimate of classical Shannon diversity index, *Q* statistic index, and Berger-Parker index, were also conducted [30]. Community richness and diversity indices were performed in MOTHUR software using (summary.single) command.

#### 2.8.2. Beta Diversity

Shared community membership and community structure were analyzed using Jaccard and Theta index, respectively. And (dist.shared) command was used to calculate beta diversity [31]. Shared community richness, shared Sobs, shared Chao 1, and shared ACE, was conducted using (summary.shared) command. The UniFrac based metrics were used to assess the similarity between two communities' membership (unifrac.unweighted) and structure (unifrac.weighted). The relation between samples was identified using principal coordinate analysis (PCoA) that was visualized to compare our samples using graphing calculator 3D program (v.6.6.2) [32, 33]. A visualized Venn diagram was illustrated using MOTHUR software using (Venn) command [25]. Dendrogram was created which described similarities between the samples using (tree.shared) command [25].

### 2.9. Statistical Analysis

For statistical analyses, PAST software (v.3.16) was used to calculate Mann–Whitney (MW) *U* test and Kruskal-Wallis (KW) rank sum test. The difference between the 2 groups was analyzed using Mann–Whitney *U* test whereas for 3 groups Kruskal-Wallis analysis of variance by ranks was used, respectively. Statistical significance was assumed at *p* < 0.05 [34]. The correlation between OTUs was assessed using Spearman correlation coefficient (SpCC) with *p* < 0.001. The command (otu.association) in MOTHUR software was used for this assessment [25]. At genus level, hierarchical dendrogram based on the Bray-Curtis distances was calculated using Vegan package (v.2.4–4) [35] and gplots package (v.3.0.1) [36] in the R programing language version (3.4.0) 2016 [37].

## 3. Results

**Figure 1:**
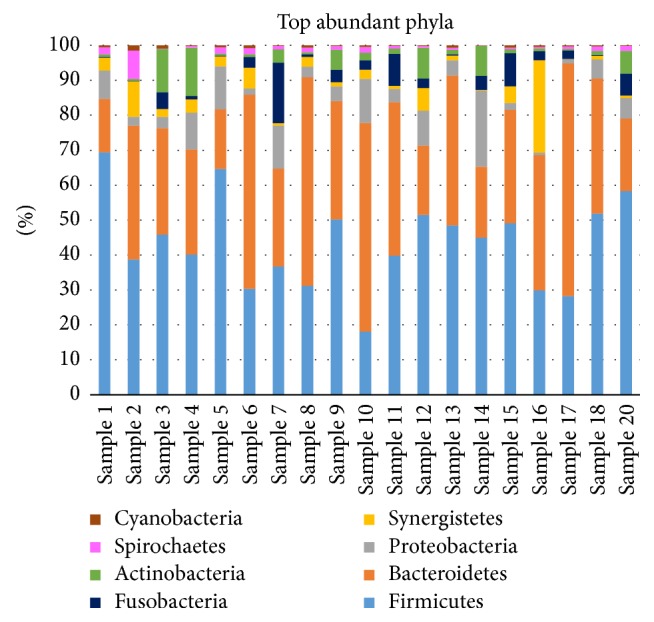
Relative abundance of the top most predominant bacterial phyla in each one of the 19 samples from the root canal content of endodontic infections. Data presented in average proportion (%) of all sequences.

**Figure 2:**
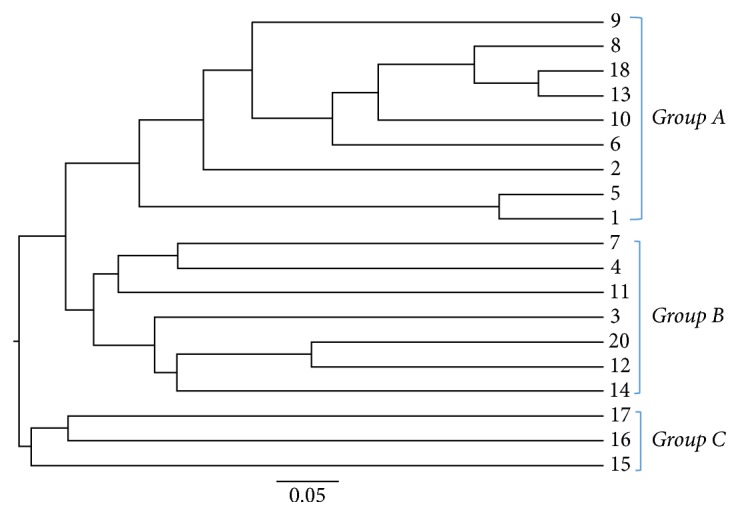
Phylogenetic tree dendrogram based on 16S rRNA gene from 19 primary endodontic patients for comparison of bacterial communities which demonstrated three separate community structure and population compositions (Groups A, B, and C).

**Figure 3:**
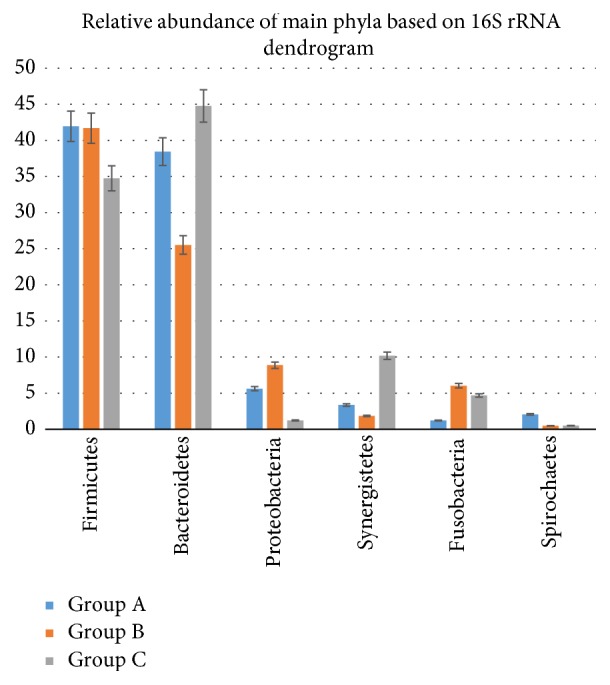
Comparison of the groups (A, B, and C) at the phylum level with error bars.

**Figure 4:**
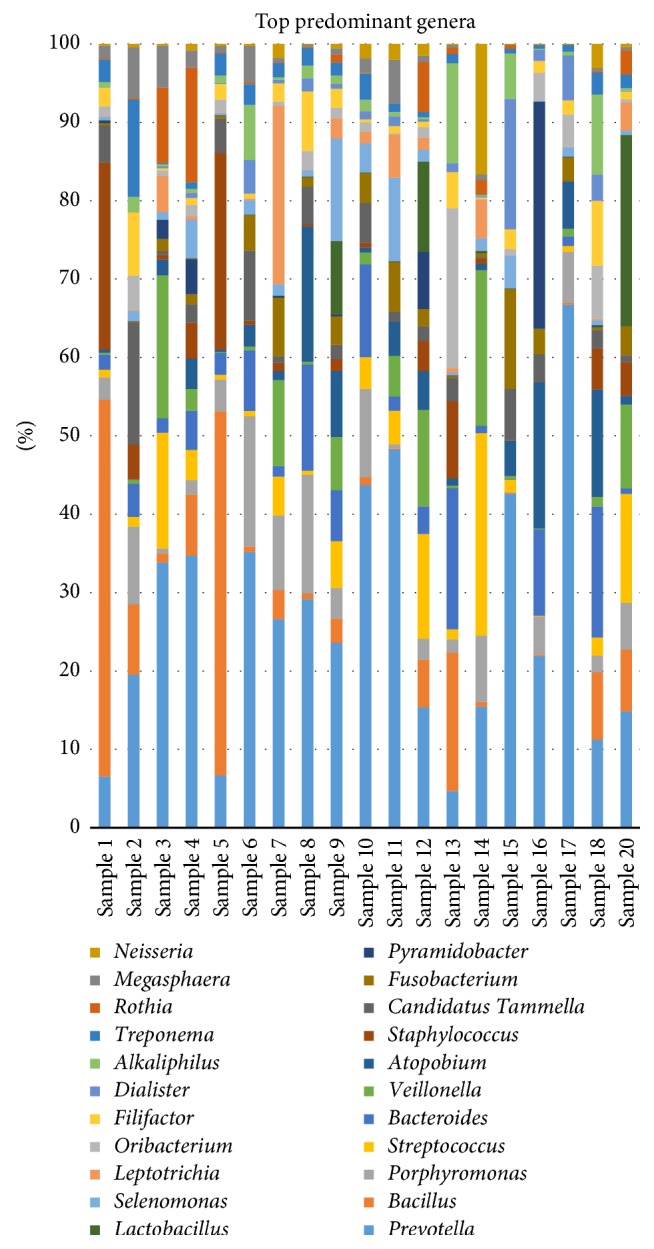
Average relative abundance of the top bacterial genera from root canal content of 19 patients with primary endodontic infections.

**Figure 5:**
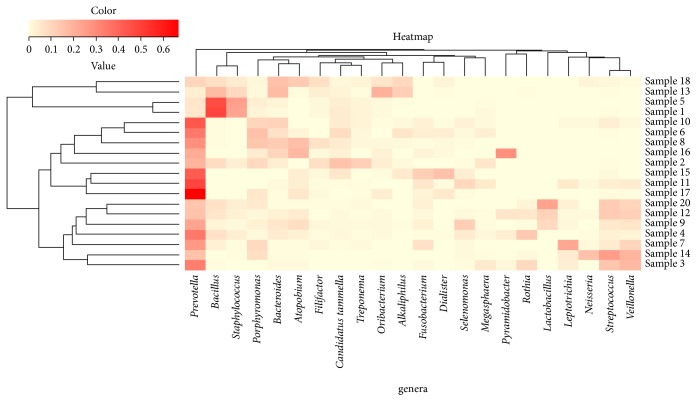
Heatmap with dendrogram at the genus level using a gradient heatmap (over 1% of the microbiome). The 22 most abundant genera were used in hierarchical clustering to evaluate the relationships between 19 samples using weighted pair clustering based on Barry-Curtis measurements. The darker the red color was the more predominant the genus was.

Paired-end sequencing on Illumina MiSeq of root canal content samples showed that all 19 cases were positive for the presence of bacterial DNA which yielded data of 2083824 raw sequences, Table 1. 1313300 unique sequences were produced from MOTHUR software analysis. Final data of 383401 sequence reads (>300 base pairs) was obtained after excluding low-quality sequence reads, preclustering, and the chimeras removal. A total of 1858 operational taxonomic units (OTUs) at 97% similarity were assigned to 26 phyla, 245 families, and 705 genera. Phyla with a representation of 0.5% and higher (relative abundance) are presented in Figure 1: Firmicutes (40.7%), Bacteroidetes (34.7%), Proteobacteria (6.1%), Synergistetes (3.9%), Fusobacteria (3.5%), Actinobacteria (3.4%), Spirochetes (1.2%), and Cyanobacteria (0.5%). Tenericutes, Chloroflexi, and Verrucomicrobia phyla were found in relatively low proportions less than 0.5%. These phyla were the most predominant, accounting for approximately 90% of the distinct OTUs found in the root canal content samples. The 19 samples were divided into three groups according to 16S rRNA dendrogram as illustrated in Figure 2. Samples 1, 2, 5, 6, 8, 9, 10, 13, and 18 (Group A) showed similarities as did samples 3, 4, 7, 11, 12, 14, and 20 (Group B). Also samples 15, 16, and 17 showed similar characteristics (Group C). Since sample code number 19 was the control sample, therefore, it was not involved in our analysis. When samples were divided into 3 groups based on 16S rRNA dendrogram, Firmicutes and Bacteroidetes were predominately found in Groups A, B, and C. The relative abundance of Firmicutes was equal in both Groups A and B, Figure 3. At genus level, the predominant genera ranked by abundance (over 1% of the microbiome) were* Prevotella* genus which accounted for 17.2% of the sequences, followed by* Bacillus* (5.1%),* Porphyromonas* (3.6%),* Streptococcus *(3.5%), and* Bacteroides* (3.2%) as shown in Figure 4.* Prevotella, Bacillus, Bacteroides, Staphylococcus*, and* Porphyromonas* were abundant in Group A, whereas* Prevotella, Streptococcus, Veillonella, Leptotrichia,* and* Lactobacillus* were abundant in Group B.* Prevotella, Atopobium, Pyramidobacter, Dialister*, and* Fusobacterium* were relatively abundant in Group C Figure 5.

The* Prevotella *genus was found with the highest proportions in all samples ranging from 13 to 53% of reads except for samples 1, 5, and 13. It was found that in samples 1, 5, and 13* Bacillus* genus was greater than* Prevotella* genus in their abundance. The species of the top three genera with their relative abundance were mentioned below. For* Prevotella* genus the main species detected were* Prevotella tannarae* (3.2%),* Prevotella intermedia* (2.3%), and* Prevotella oris* (2.1%) of all sequences. On the other hand, the major species of* Bacillus* were* Bacillus firmus* (2.4%),* Bacillus siralis* (1%), and* Bacillus horneckiae* (0.4%).* Porphyromonas endodontalis* (2%) and* Porphyromonas gingivalis* (0.8%) were the major species in* Porphyromonas *genus. Classification of sequences by alignment with reference database SILVA at 97% similarity revealed that there were sequences that were not aligned to reference sequence at different taxonomic levels. At the genus or species levels, the number of unclassified sequences increased by aligning to higher taxonomic levels. At the phylum level the mean percentage of sequences not assigned to any phyla was 5.7% while at the class, order, family, genus, and species level the mean percentage of unclassified sequences was 5.93%, 7.32%, 15.88%, 16.98%, and 36.55% respectively.

### 3.1. Alpha Diversity

**Figure 6:**
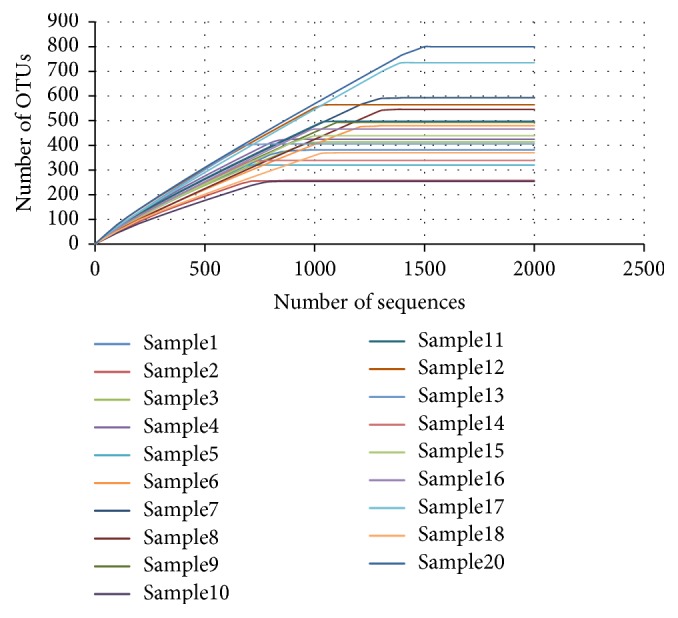
Rarefaction curves of 16S rRNA gene sequences for each sample calculated for OTUs at 97% similarity. Vertical axis shows operational taxonomic units, and horizontal axis shows the number of samples sequenced. OTUs = operational taxonomic units.

Table 2 depicts data from community richness calculations. A predicted mean of 6161 and 16966 distinct OTUs per root canal content sample from Chao and ACE nonparametric measures of richness had been revealed, respectively. In addition, Table 3 depicts data from community diversity calculations. The good estimator detected > 90% coverage for the overall data, meaning that only 10 OTUs would be found if 100 additional sequences have been analyzed. Moreover, the rarefaction curve shape showed that bacterial richness in the root canal content samples was completely revealed by the number of sequences analyzed, Figure 6. Alpha diversity indices at 97% similarity were calculated using random resampling (2000 sequences).

### 3.2. Beta Diversity

The mean value of pairwise comparisons between different samples was 0.96 for Jaccard and 0.86 for Theta. These results revealed more similarity between samples.  Table 4 displayed data on shared observed species for OTUS (at genus level) and shared Chao index.

### 3.3. Grouping of Samples and Its Statistical Results

**Figure 7:**
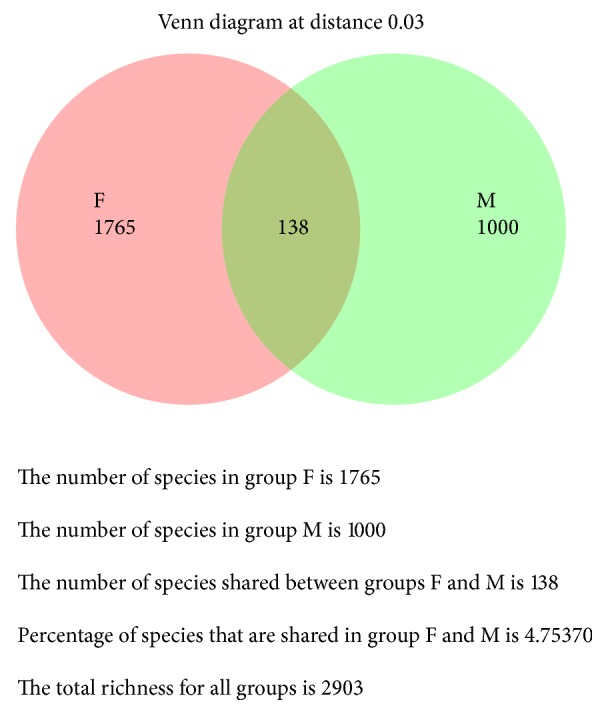
Venn diagram illustrated the number of shared OTUs between males and females at 97% similarity. Colored circles represented each sex and intersection part between circles represented the number of shared OTUs.

**Figure 8:**
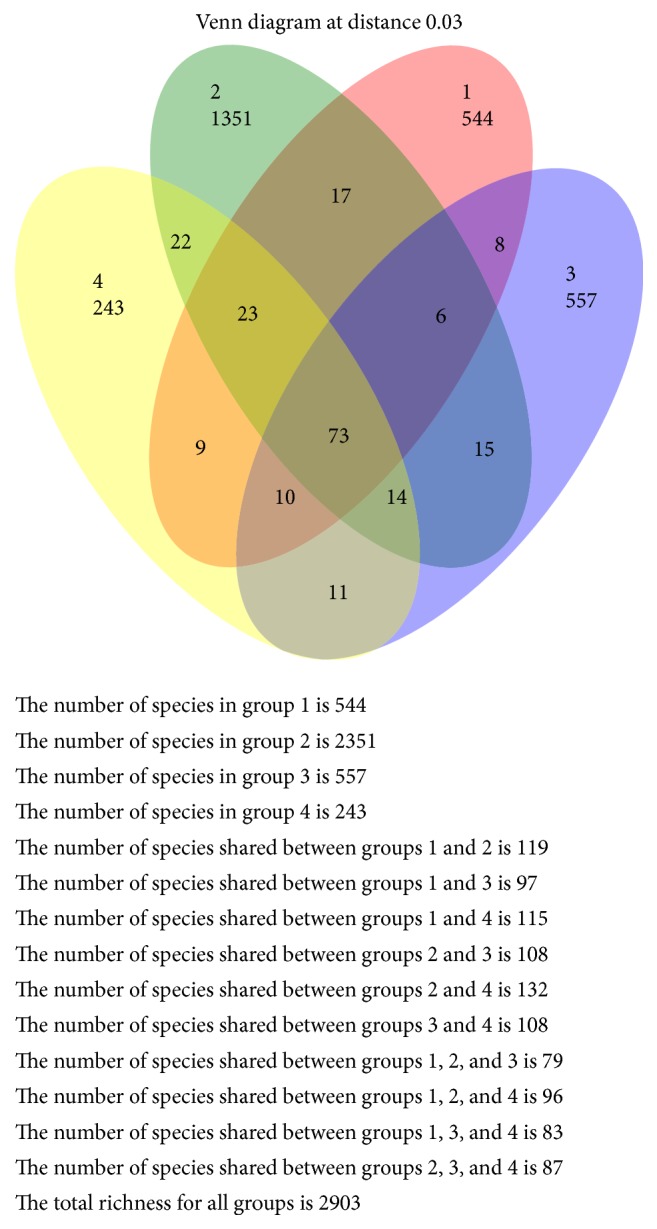
Venn diagram illustrated the number of shared OTUs according to four age groups at 97% similarity. Colored circles represented each age group and intersection part between circles represented the number of shared OTUs.

Samples were classified into three categories A, B, and C to assist a comparative analysis. The results of using Kruskal-Wallis (KW) rank sum test to compare between 3 groups were presented in Table 5. Mann–Whitney *U* test was used to compare between 2 groups. Comparison between male and female groups revealed significant differences only for* Prevotella* (*p* = 0.01) and* Bacteroides *(*p* = 0.005) genera. The relative abundance of* Prevotella *genus in males (14.3%) was higher than females (3.8%) whereas, in the* Bacteroides* genus, the relative abundance was greater in females (4%) than males (0.6%). On the other hand, there was no significant difference between the four age groups using Kruskal-Wallis (KW) rank sum test. Illustrated Venn diagram was represented for sex and age groups in Figures 7 and 8.

**Figure 9:**
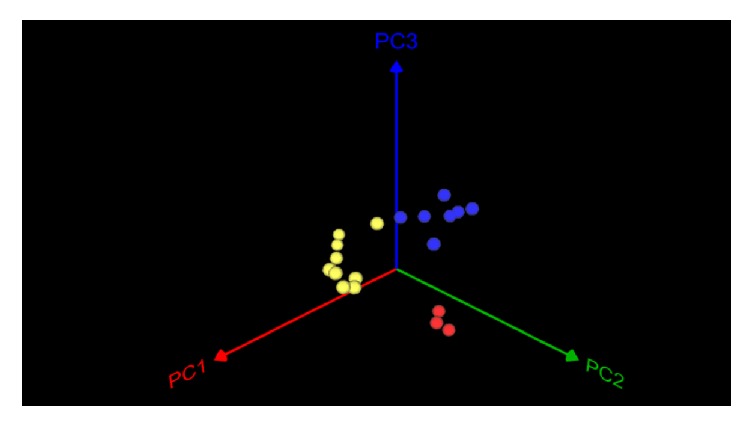
A three-dimensional plot of weighted UniFrac based principal coordinate analysis (PCoA). Colors represented the three groups based on 16S rRNA dendrogram. Yellow, blue, and red balls resembled Groups A, B, and C respectively. Plot was created using the pairwise weighted UniFrac distances (where PC1 is variability at 25.57%, PC2 is variability at 14.69%, and PC3 is variability at 11.44%). Samples from the same group cluster more closely together.

Relationships between various samples were evaluated using UniFrac based principal component analysis (PCoA). Principal coordinate analysis based on UniFrac revealed that clustering of samples was according to grouping of the 16S rRNA dendrogram rather than samples, Figure 9. Correlations between various members of the endodontic microbiome at different taxonomic levels were assessed using Spearman correlation coefficient (SpCC). There were 8829 (*r* = >0.6, *p* value < 0.001) significant positive correlations which expressed as a positive relationship between the two variables. The strongest correlations (*r* = 0.82) were detected between the most abundant phyla in all samples: Firmicutes, Bacteroidetes, Actinobacteria, Synergistetes, and Fusobacteria. The majority of samples showed positive SpCC mainly between OTUs which belonged to Lachnospiraceae family. While at genus level, the most significant positive SpCC was detected between OTUs assigned to* Oribacterium *and* Atopobium *genera. In parallel, minor samples resulting with negative SpCC (*r* = <−0.6, *p* value < 0.05) between OTUs were related to Fusobacteriaceae and Spirochaetaceae families. The significant negative SpCC showed between OTUs was related to* Fusobacteria, Treponema, Bacillus, *and* Prevotella* genera.

## 4. Discussion

Primary endodontic infection is caused by microorganisms that invade and colonize the necrotic pulp tissue. It is distinguished by a mixed society prominently dominated by anaerobic bacteria [38, 39]. There were variations at the bacterial profiles of the endodontic microbiota from person to person [39]. This means that each individual is hosted by unique endodontic microbiota in terms of species richness and abundance. This suggests that primary endodontic infection has a heterogeneous etiology where no single species can be considered a major endodontic pathogen [19]. Primary endodontic infection is caused by multiple bacterial combinations which were evident by high numbers of different taxa that have been detected [22].

Culture-independent molecular biology methods had overcome many limitations of culture techniques and have been used recently for microbial characterization in endodontic research [38, 40–42]. Root canal microbiome was investigated by several methods. Many studies used extracted teeth subjected to cryogenic grinding approach where PCR products from extracted DNA were separated by denaturing gradient gel electrophoresis to obtain fingerprinting of bacterial communities [8, 43]. Other studies also used extracted teeth but extracted DNA was subjected to pyrosequencing analysis [44, 45] or Illumina sequencing [46]. Moreover, apical periradicular lesions obtained during apical surgery have been used for sampling root canal [47]. Our study used files and paper points for sampling as other studies did, because it is a simple and easy method [9, 17, 19, 22, 23, 48, 49]. In addition, another advantage of this method of collection is that it does not require extracted tooth to be processed or teeth subject to root-end resection during apical periradicular surgery. The collection method however had limitations for the characterization of the total endodontic microbiome due to anatomical variations between the patients such as fins, lateral canals, dentinal tubules, and isthmuses [19]. There is no ideal approach for endodontic microbiological sampling without limitations. The method of sample collection should be adequate to the purpose of the study in the research design. The results of the Illumina MiSeq sequencing have showed that the root canal can harbor a highly diverse microbiome [19]. These previous studies used pyrosequencing technique [9, 17, 19, 20, 44, 45, 49] and their sample sizes were 20, 18, 7, 20, 17, 10, and 10, respectively. On the other hand, these two studies [22, 46] with sample sizes of 10 and 12 patients were utilized in Illumina sequencing platform. Therefore, the use of 19 participants was suitable for our study aim.

The analysis of the 16S ribosomal RNA gene sequence is commonly used in metagenomic studies. The 16S gene is universal in all bacteria as it gathers the advantage of being long enough to provide high information but, also at the same time, short in length that it can be easily sequenced. The 16S rRNA gene is composed of around 1500 base pairs. It contains nine variable and conserved regions in such a manner that conserved region is followed by variable region. Phylogenetic classification is performed using variable regions of 16S rRNA. To debate, the reason of choosing variable region depends on various factors such as objectives of the experiment, experimental design, and sample type [50]. Since the bacterial diversity is largely wide, it may be recommended that more than one variable region be targeted. This increases the specificity, accuracy, and sensitivity of the 16S gene study [20]. Our study used V3 and V4 regions. An experimental study revealed that targeting the V3 and V4 regions produced high quality of sequenced data [51]. In addition, these regions were recommended by Illumina protocol manual [52]. To ensure that microbial classification is of high quality, our study targeted two variable regions in order to achieve pair-ended reads of approximately 490 bp.

The main bacterial phyla detected in the primary endodontic infection were Firmicutes and Bacteroidetes, which together represented about three-quarters of all sequences obtained. The next most abundant phyla were Proteobacteria and Synergistetes. In previous studies using culturing and molecular methods, Firmicutes was the most abundant phylum in endodontic infection [5, 6]. In other previous studies which used pyrosequencing, there was a difference in the most dominated phylum. Some studies showed Bacteroidetes [9, 17, 23] as the major phylum whereas other studies demonstrated Firmicutes [19, 22, 45, 49] and Proteobacteria [44, 46, 47], respectively. The difference in the phylum results may be due to different sampling method, different clinical expressions and interventions, analytical artifacts during PCR, and filtering sequences or identification. It is important to take into consideration the fact that most previous studies used pyrosequencing whereas few studies used Illumina sequencing and our study was one of these studies. Our data was in agreement with those provided by Vengerfeldt et al. [22] which revealed Firmicutes and Bacteroidetes as the top two main phyla. We would like to focus on the fact that this study was the first study which characterized endodontic infection using next generation sequencing (MiSeq Illumina) in the Middle East, Egypt. As a result, this study gains the benefit of simplicity of sampling procedure and millions of reads were generated per instrument run with less cost and more depth coverage in comparison to pyrosequencing.

Due to variations in the abundance of OTUs in the groups, the groups appeared to show different stages of endodontic infections. The* Lactobacillus* genus was abundant in Group B, which indicated the initial stage of endodontic infection [53, 54]. Bacteroidetes and* Porphyromonas* were abundant in Group A. Group A might appear in another stage of infection. In a previous study of Gomes et al. [55],* Tannerella forsythia *was found to be a member of the* Bacteroides* genus which is most likely recovered from an acute dental abscess.* Tannerella forsythia *species was associated with tenderness to percussion [55]. Also* Porphyromonas* was present in high levels in Group A where in a previous study* P. endodontalis* and* P. gingivalis* were associated with the presence of a sinus tract and abscess formation [56]. Gomes et al. [57, 58] recognized that the microbiota found in teeth with a sinus was predominantly mixed. Group C included* Fusobacterium* species with high abundance in comparison to the other two groups.* Fusobacterium* was recognized with more severe endodontic infection with pain or history of pain and was linked to polymicrobial infections due to bacterial synergism [48, 59, 60].* Prevotella* genus was present in high levels in all the three groups but in higher proportion in Group C followed by Group B and then Group A.* Prevotella* species are strictly anaerobes and highly virulent species which was the reason for their high abundance [61]. This might also give indication that higher abundance of* Prevotella* is related to the degree and stage of infection. Jung et al. [62] indicated that the degree of severity of an endodontic infection is related not only to the presence of pathogens but also to the numbers of those organisms in the infected site. Our study was in agreement with the previous studies results which showed that the species* Porphyromonas gingivalis, Porphyromonas endodontalis, Prevotella intermedia, *and* Prevotella nigrescens* were abundant in endodontic infection [61, 63, 64].* Treponema* genus was from the top twenty genera found in this study. Our research is in line with the findings of Siqueira Jr. and Rôças [39] which revealed that* Treponema denticola*,* Treponema socranskii*,* Treponema maltophilum*,* Treponema amylovorum*, and* Treponema medium* were from the most prevalent species in* Treponema*.

This study identified 1858 different OTUs with >3% dissimilarity, belonging to 705 genera. When identifying each sample for its highest dominated genera, it revealed that 9 samples were dominated by* Prevotella *and only 2 samples were dominated by* Bacillus* genus. There were 8 samples dominated by bacteria that could not be identified for the genus level (unclassified). This was the number of genera that were found by these studies: Hong et al. [9] specified 133, Tzanetakis et al. [23] 347, and Anderson et al. [26] 525. The differences between these studies are linked to the number of samples examined, the depth of sequencing, the analytical methods for identification, and the selected region sampled (whole main canal in the paper point approach versus apical canal in the cryopulverization approach) [46].

Acidobacteria, Chloroflexi, and Cyanobacteria are phyla which exist in soil, wastewater plants, and water. When next generation sequencing instruments were invented, these phyla were detected in endodontic infections which were not previously known to be found in endodontic infections [17]. Most of these phyla were found in relatively low abundance and were unnoticed in previous studies characterizing endodontic communities.

Regarding composition level, heatmap examination revealed a remarkable interindividual variability in the bacterial communities' composition. Each patient harbored unique endodontic microbiome for abundance and richness of species. In fact, microbiota composition varied between individuals having the same disease which means that endodontic infections were caused by heterogeneous etiology. In other words, similar disease resulted from multiple communities. Regardless of this interindividual variance, according to the pairwise weighted UniFrac distances, samples tended to cluster based on 16S rRNA dendrogram.

Alpha diversity analysis indicated that the diversity is almost investigated as illustrated in the rarefaction curves but we must take into consideration the fact that using paper points and file in our sample as a collection method may not be the most favorable method to determine the whole microbiome in lateral canals, dentinal tubules, and isthmus. This suggests that the whole bacterial diversity in primary endodontic infections may be higher than actually identified.

Our results from sequencing analyses indicated that primary endodontic infections were dominated by both anaerobic and facultative anaerobic species. It was previously thought that primary endodontic infections were dominated by anaerobic species while facultative anaerobes were more predominant in secondary infections [42, 65]. Recently, next generation sequencing studies have demonstrated mixed results of anaerobic and facultative anaerobic species [66].

It is important to characterize the composition of the endodontic microflora because this may be related to the different clinical presentations or stages of development of an endodontic infection as well as its responses to different treatments [19]. Large numbers of OTUs were found at low abundance at our study. This ensures the power of Illumina analysis to detect the bacterial communities in primary endodontic infection [22]. However, it is difficult to determine the role of each bacterium in the community [18]. But it is clearly seen that even low abundant species play an important role in the endodontic oral microbiome [18]. Shifts in environmental conditions may result in the fact that members with low abundance may become dominant as a response to these changes [49]. Our study demonstrated that Illumina MiSeq platform sequencing technology allowed us to identify low abundant bacteria in infected root canals and also detect bacterial diversity which was not previously known in endodontic microbiota. As a result, more accurate estimation of species abundance and prevalence can be determined in the endodontic microbial community [12]. Endodontic microflora found in low proportions can even occupy critical niches within a complex microbial community, and so it is important to detect these members to maintain the stability and virulence of a microbial community [12]. The utilization of MiSeq Illumina sequencing can broaden our understanding of the pathogenesis of endodontic infections and has the power to improve treatment outcomes.

Next generation sequencing instruments are unable to differentiate between living and dead microorganisms and so all genetic material is measured [15]. This may be considered as an overestimate of bacterial count because even after cell death DNA can persist for a period of one year [67]. There is an argument that assessing living and dead microorganisms is essential because these microorganisms may have been dominant in the early stages of disease [47]. In addition, there has been an argument concerning the quality of taxonomic identification since that next generation sequencing used short sequencing reads [50]. But recently, NGS compared to early NGS are able to sequence different read lengths ranging from 50 bp up to 700 bp [66]. There are other factors which can affect the sequencing results such as sequencing errors, primer selection bias, chimera formation, interpretation of the huge data produced by bioinformatics programs, and PCR conditions [68, 69].

## 5. Conclusion

This study revealed that microbiota of endodontic infection with periapical lesions had high polymicrobial communities. To fully understand the etiology of endodontic disease, a further and deeper host-microbiome analyses should be performed. Since that the results of our community largely varied from case to case, therefore this suggests that this disease is characterized by multispecies bacterial communities having a heterogeneous etiology.

## Figures and Tables

**Table 1:** Sequence analysis for the generated reads and the remaining reads after preprocessing, filtration, preclustering, and chimeras removal.

Sample code	Total reads	Reads passing quality filtering	Number of unique reads	Total number of reads before preclustering	Total number of reads after preclustering	Number of chimeras	Number of reads after chimeras removal	Percentage of chimeras sequence
1	130378	102374	69001	52390	22726	4334	18392	19.10%
2	99713	78230	52970	43726	20319	6216	14103	30.60%
3	148927	117143	83994	65789	30793	7323	23470	23.80%
4	136015	107981	73508	52409	24382	5070	19312	20.80%
5	91410	72590	54671	43282	22740	4037	18702	17.80%
6	159563	124396	81290	65593	29018	7503	21515	25.90%
7	179597	140847	94637	72983	32522	6942	25580	21.30%
8	153991	121551	76195	59673	26540	8457	18083	31.90%
9	140290	108819	77616	59922	27164	5045	22119	18.60%
10	93517	75671	50873	40472	17933	4568	13365	25.50%
11	153126	120768	91730	75798	41305	18169	23136	44%
12	159655	129432	95261	67017	31948	6394	25554	20%
13	72461	57647	40291	32138	16499	5210	11289	31.60%
14	98370	77375	56593	46254	22690	5828	16862	25.70%
15	126414	99959	65761	52345	24373	7679	16694	31.50%
16	128433	101274	65627	52214	24255	9275	14980	38.20%
17	202643	155799	107509	84438	42083	18983	23100	45.10%
18	183073	144386	96114	73998	33752	8485	25267	25.10%
20	188766	147582	107487	84994	41383	9505	31878	23%
Average	139281.2	109674.9	75848.84	59233.42	28022.37	7843.32	20179	27%

**Table 2:** Community richness indices: Sobs, Chao, ACE, and Jackknife.

Sample	Sobs	Chao	ACE	Jackknife
1	382	4815.077	12727.76	2609.788
2	258	3575.143	7171.851	15878.39
3	414	3142.682	8772.472	2691.427
4	425	3297.435	7720.03	2891.89
5	320	4077.091	16280.43	3722.471
6	480	7129.714	22638.01	3919.938
7	593	8777	21779.88	4134.895
8	546	10491.08	35406.91	4547.667
9	494	4541.652	11181.97	3302.81
10	254	1773.4	5302.989	1317.182
11	498	4931.143	10405.96	3422.119
12	565	5020.37	13945.89	5031.037
13	406	5131.067	23696.3	3136.874
14	339	3190.867	8166.598	2103.402
15	439	10414	23551.97	3477.703
16	466	10524.33	19000.8	4022.645
17	735	11259.55	37465.82	5812.206
18	370	3902.5	9036.737	2307.078
20	800	11067.44	28107.83	6655.509

Sobs: number of observed OTUs (at genus level); Chao: Chao 1 richness estimate; ACE: abundance based coverage richness estimate; Jackknife: Jackknife estimator.

**Table 3:** Community diversity indices: Coverage, Simpson, Invsimpson, Shannon, Npshannon, *Q*stat, and Berger-Parker.

Group	*N*seqs	Coverage	Simpson	Invsimpson	Shannon	Npshannon	*Q*stat	Berger-Parker
1	2000	0.99	0.05	20.46	4.64	5.29	253.91	0.19
2	2000	0.90	0.03	38.10	4.52	4.90	160.14	0.09
3	2000	0.94	0.02	65.38	5.07	5.51	265.46	0.05
4	2000	0.96	0.02	59.33	5.21	5.78	278.44	0.08
5	2000	0.97	0.03	30.90	4.69	5.32	214.96	0.13
6	2000	0.94	0.03	29.92	4.80	5.33	321.00	0.14
7	2000	0.99	0.02	60.74	5.32	5.85	392.41	0.08
8	2000	0.91	0.07	14.44	4.53	5.28	375.82	0.24
9	2000	0.91	0.02	49.64	5.12	5.65	327.49	0.09
10	2000	0.92	0.08	12.21	3.90	4.44	164.47	0.23
11	2000	0.99	0.02	56.74	5.23	5.77	326.05	0.07
12	2000	0.92	0.01	119.61	5.65	6.20	372.94	0.04
13	2000	0.95	0.06	17.85	4.90	5.85	282.05	0.23
14	2000	0.99	0.02	43.05	4.88	5.44	221.45	0.09
15	2000	0.99	0.04	28.08	4.83	5.45	293.59	0.15
16	2000	0.97	0.03	31.07	4.91	5.56	313.06	0.12
17	2000	0.95	0.02	45.51	5.46	6.20	506.39	0.11
18	2000	0.96	0.08	13.15	4.35	4.88	236.60	0.26
20	2000	0.95	0.01	114.42	5.86	6.44	534.52	0.05

*N*seqs: number of sequences in the sample; Simpson: Simpson diversity index; Invsimpson: inverse Simpson index (1/*D*); Shannon: Shannon diversity index; Npshannon: nonparametric estimate of classical Shannon diversity index; *Q*stat: *Q* statistic index; Berger-Parker: Berger-Parker index.

**Table 4:** Shared OTUs (bold) and shared Chao index (italicized) between each pair of the dental root canal content samples.

	Sample 1	Sample 2	Sample 3	Sample 4	Sample 5	Sample 6	Sample 7	Sample 8	Sample 9	Sample 10	Sample 11	Sample 12	Sample 13	Sample 14	Sample 15	Sample 16	Sample 17	Sample 18	Sample 20
Sample 1	-	**41**	**34**	**36**	**41**	**30**	**38**	**19**	**39**	**25**	**18**	**42**	**23**	**17**	**14**	**13**	**15**	**36**	**43**
Sample 2	*57.04*	-	**29**	**34**	**42**	**30**	**33**	**25**	**34**	*47.50*	*110.00*	*90.38*	*26.50*	*86.50*	*66.50*	*36.50*	*36.65*	*80.50*	*101.11*
Sample 3	*68.33*	*112.64*	-	**44**	**32**	**24**	**43**	**23**	**46**	*40.58*	*92.50*	*92.01*	*35.77*	*110.10*	*54.75*	*33.00*	*31.25*	*62.71*	*77.54*
Sample 4	*57.85*	*84.22*	*77.33*	-	**37**	**32**	**48**	**25**	**49**	*42.10*	*57.42*	*80.74*	*50.06*	*88.07*	*49.75*	*53.13*	*54.00*	*74.97*	*85.3125*
Sample 5	*63.27*	*80.57*	*72.85*	*83.75*	-		**31**	**26**	**37**	*43.90*	*78.00*	*62.87*	*40.64*	*73.00*	*72.00*	*42.00*	*47.00*	*72.28*	*84.68*
Sample 6	*46.68*	*56.58*	*49.23*	*41.33*	*67.50*	-	**34**	**29**	**41**	*46.89*	*49.58*	*49.50*	*30.90*	*70.75*	*33.61*	*44.50*	*39.90*	*57.08*	*53.71*
Sample 7	*89.63*	*63.42*	*76.10*	*96.48*	*55.50*	*39.26*	-	**26**	**47**	*45.64*	*77.17*	*91.47*	*69.33*	*71.33*	*43.50*	*38.00*	*36.86*	*58.58*	*77.67*
Sample 8	*29.25*	*46.17*	*39.42*	*43.48*	*64.00*	*41.60*	*36.50*	-	**35**	*40.88*	*66.33*	*49.42*	*42.38*	*31.83*	*35.83*	*29.00*	*39.23*	*53.76*	*38.79*
Sample 9	*63.79*	*80.93*	*63.71*	*105.64*	*76.76*	*47.14*	*65.66*	*73.64*	-	*39.82*	*85.21*	*101.58*	*71.64*	*79.50*	*57.08*	*40.00*	*36.05*	*125.85*	*80.58*
Sample 10	*48.79*	**25**	**27**	**30**	**23**	**26**	**34**	**22**	**30**	-	**32**	**42**	**23**	**26**	**20**	**19**	**19**	**29**	**39**
Sample 11	*33.00*	**25**	**46**	**34**	**24**	**38**	**39**	**31**	**48**	*44.95*	-	**49**	**21**	**41**	**34**	**22**	**31**	**37**	**49**
Sample 12	*67.14*	**36**	**65**	**56**	**35**	**40**	**56**	**27**	**60**	*77.94*	*115.39*	-	**32**	**52**	**31**	**35**	**32**	**47**	**68**
Sample 13	*38.17*	**20**	**22**	**31**	**25**	**20**	**25**	**22**	**32**	*44.07*	*34.04*	*60.35*	-	**18**	**19**	**18**	**14**	**37**	**28**
Sample 14	*46.00*	**21**	**46**	**34**	**20**	**25**	**31**	**16**	**37**	*64.75*	*68.48*	*124.23*	*33.70*	-	**20**	**12**	**16**	**29**	**50**
Sample 15	*28*	**17**	**26**	**27**	**21**	**29**	**23**	**25**	**35**	*50.70*	*57.58*	*54.11*	*42.14*	*36.83*	-	**18**	**23**	**29**	**26**
Sample 16	*16*	**16**	**18**	**28**	**15**	**23**	**23**	**20**	**23**	*29.25*	*41.50*	*63.43*	*22.67*	*33.50*	*26.50*	-	**25**	**25**	**23**
Sample 17	*21*	**18**	**21**	**25**	**19**	**30**	**27**	**28**	**30**	*20.20*	*37.92*	*45.65*	*16.15*	*34.83*	*25.89*	*40.70*	-	**28**	**27**
Sample 18	*84.1*	**37**	**37**	**43**	**38**	**39**	**35**	**35**	**54**	*45.00*	*71.04*	*63.49*	*74.19*	*61.00*	*50.42*	*31.00*	*39.75*	-	**49**
Sample 20	*70.4*	**51**	**48**	**51**	**45**	**34**	**48**	**27**	**54**	*59.00*	*81.24*	*114.24*	*42.02*	*111.21*	*102.14*	*48.70*	*38.42*	*92.80*	-

**Table 5:** ^*∗*^Derived from Kruskal-Wallis sum rank test. Bolded numbers are statistically significant (*p* < 0.05).

Genus	Relative abundance (%)			*p* value (KW)^*∗*^
	Group A	Group B	Group c	
*Prevotella*	11.37	17.74	33.60	0.05
*Bacillus*	8.81	2.42	0.12	**0.04**
*Porphyromonas*	4.40	2.74	3.05	0.4
*Streptococcus*	1.05	7.94	0.60	**0.004**
*Bacteroides*	4.75	1.31	3.27	**0.04**
*Veillonella*	0.70	7.75	0.38	**0.002**
*Atopobium*	2.58	1.67	7.63	0.12
*Staphylococcus*	4.61	1.35	0.04	**0.04**
*Candidatus *Tammella	3.35	0.69	2.50	**0.02**
*Fusobacterium*	0.93	2.17	4.69	**0.04**
*Pyramidobacter*	0.06	1.22	7.71	0.3
*Lactobacillus*	0.61	3.76	0.04	0.08
*Selenomonas*	1.39	2.05	1.33	0.5
*Leptotrichia*	0.31	3.90	0.03	**0.002**
*Oribacterium*	2.15	0.41	2.23	0.08
*Filifactor*	2.19	0.55	1.47	**0.04**
*Dialister*	0.73	0.29	5.85	**0.03**
*Alkaliphilus*	2.10	0.23	1.53	**0.004**
*Treponema*	2.10	0.58	0.50	**0.02**
*Rothia*	0.16	3.12	0.12	0.1

## Data Availability

The sequenced data obtained from the Illumina MiSeq sequencing had been submitted to NCBI BioProject (https://www.ncbi.nlm.nih.gov/bioproject) under Accession no. PRJNA388365. Biosample submission at the NCBI BioProject (https://www.ncbi.nlm.nih.gov/biosample) was accepted under Accession nos. SAMN07174627, SAMN07174628, SAMN07174629, SAMN07174630, SAMN07174631, SAMN07174887, SAMN07174888, SAMN07174889, SAMN07174986, SAMN07174987, SAMN07174988, SAMN07174989, SAMN07174990, SAMN07174991, SAMN07175002, SAMN07175155, SAMN07175156, SAMN07175157, and SAMN07175158. The sequences from the 19 endodontic samples are available at the NCBI Sequence Read Archive (SRA) under Accession no. SRP108240.

## Abbreviations

NGS: Next generation sequencing
OTUs: Operational taxonomic units
MW*U* test: Mann–Whitney *U* test
KW rank sum test: Kruskal-Wallis rank sum test
PCR: Polymerase chain reaction
PCoA: Principal coordinate analysis
NaOCl: Sodium hypochlorite
UniFrac: Unique fraction
rRNA: Ribosomal RNA
DNA: Deoxyribonucleic acid.
